# RNA Microarray-Based Comparison of Innate Immune Phenotypes between Human THP-1 Macrophages Stimulated with Two BCG Strains

**DOI:** 10.3390/ijms23094525

**Published:** 2022-04-20

**Authors:** Gabriela Molina-Olvera, Claudia I. Rivas-Ortiz, Alejandro Schcolnik-Cabrera, Antonia I. Castillo-Rodal, Yolanda López-Vidal

**Affiliations:** Programa de Inmunología Molecular Microbiana, Departamento de Microbiología y Parasitología, Facultad de Medicina, Universidad Nacional Autónoma de México (UNAM), Ciudad de México 04510, Mexico; gabriela.molina@uvmnet.edu (G.M.-O.); rivasorclaudia@ciencias.unam.mx (C.I.R.-O.); adrian.alejandro.schcolnik.cabrera@umontreal.ca (A.S.-C.)

**Keywords:** THP-1 cells, BCG strains, RNA microarrays, phagocytosis, innate immune responses

## Abstract

Currently, the only available vaccine against tuberculosis is *Mycobacterium bovis* Bacille Calmette-Guérin (BCG). Pulmonary tuberculosis protection provided by the vaccine varies depending on the strain, the patient’s age and the evaluated population. Although the adaptive immune responses induced by different BCG strains have been widely studied, little conclusive data is available regarding innate immune responses, especially in macrophages. Here, we aimed to characterize the innate immune responses of human THP-1-derived macrophages at the transcriptional level following a challenge with either the BCG Mexico (M.BCG) or Phipps (P.BCG) strains. After a brief in vitro characterization of the bacterial strains and the innate immune responses, including nitric oxide production and cytokine profiles, we analyzed the mRNA expression patterns and performed pathway enrichment analysis using RNA microarrays. Our results showed that multiple biological processes were enriched, especially those associated with innate inflammatory and antimicrobial responses, including tumor necrosis factor (TNF)-α, type I interferon (IFN-I) and IFN-γ. However, four DEGs were identified in macrophages infected with M.BCG compared to P. BCG. These findings indicated the proinflammatory stimulation of macrophages induced by both BCG strains, at the cytokine level and in terms of gene expression, suggesting a differential expression pattern of innate immune transcripts depending on the mycobacterial strain.

## 1. Introduction

Tuberculosis (TB) is a chronic infectious disease caused by *Mycobacterium tuberculosis* (Mtb), a bacterium that killed almost 70% of infected patients in the predrug era [1]. Nearly 25% of the global population is infected by Mtb, and during 2019, the incidence of TB was estimated at 10 million patients, and more than 1.2 million deaths were directly caused by TB [2]. The only currently approved vaccine for TB prevention is the attenuated form of *Mycobacterium bovis*, the bacillus Calmette-Guérin (BCG). This vaccine provides approximately 80% protection against the meningeal and disseminated TB forms, but the protective effects of the vaccine against pulmonary TB in infants or pulmonary TB reactivation in adults remain controversial [3,4]. Although significant progress has been made toward understanding the adaptive immune response induced by varying different strains of the BCG vaccine [5,6], evidence regarding the innate response is lacking.

Mtb is an intracellular bacterium that is phagocytosed by the alveolar macrophage. Once internalized, the bacterium creates a niche that inhibits its degradation to allow its survival, growth and replication [7]. The BCG vaccine requires a bacterial attenuation process to reduce *Mycobacterium* virulence [8]. However, this approach is also related to mutations that can decrease the immune system stimulation and shorten the period of protection [9,10]. Due to genomic heterogeneity among BCG strains, the correlation between phenotypic characteristics and the conferred immune protection level is complicated, and the ability of BCG to induce an adaptive immune response is not necessarily linked to its protective efficacy [11,12]. The development of an improved vaccine requires increased knowledge regarding the characteristics of the innate immune response stimulated by the BCG vaccine. During the first critical phase of human infection with BCG, the internalization of the bacteria into macrophages occurs, and the cascade of innate immune responses that follow must be more clearly described to provide better options for the vaccine development process.

The RNA microarray technology is an approach that has been used to study biological functions and pathways at the transcriptional level, probing RNA with target molecules to analyze the relative gene expression in the sample of interest [13,14]. Due to the complexity of crosstalk and interactions that occur between the BCG bacteria and the host macrophage [15], the global evaluation of the macrophage response using RNA microarrays would facilitate an in-depth description of early innate immune responses generated by macrophages in response to the infection. Monocyte-derived macrophages differentiate into specific phenotypes, driven by key cytokines in the microenvironment and receptor-level interactions [16]. One factor that affects this process is the Toll-like receptor (TLR) family, which activates multiple signaling cascades and plays fundamental roles in the host defense mechanisms by inducing the expression of inflammatory molecules, including tumor necrosis factor (TNF)-α, the proinflammatory cytokine interleukin (IL)-12 and nitric oxide (NO), which contribute to pathogen destruction [17,18].

Previous work by our group using a mouse model of progressive pulmonary tuberculosis induced by the MtbH37Rv strain showed differences in protection associated with different strains of the BCG vaccines. The BCG Phipps (P.BCG) strain provided the greatest protection, whereas the BCG Mexico (M.BCG) strain induced a moderate immune response [19]. In this study, we aimed to study the transcriptional responses induced in a human macrophage derivate from monocytes of the THP-1 cell line. The usefulness of this cell line to test immune modulation [20] and specifically BCG responses [21,22], following infection with either P.BCG or M.BCG, has been previously reported, We briefly performed a characterization of the innate immune responses in vitro at different hours and selected 24 h to evaluate with RNA expression microarrays the differentially expressed genes (DEGs) in infected macrophages with each BCG strain. Finally, both functional and pathway enrichment analyses were performed to describe the innate immune responses induced by BCG-infected macrophages.

## 2. Results

### 2.1. Characterization of the THP-1 Cells Infected with the BCG Mexico or Phipps Strains

**Figure 1:**
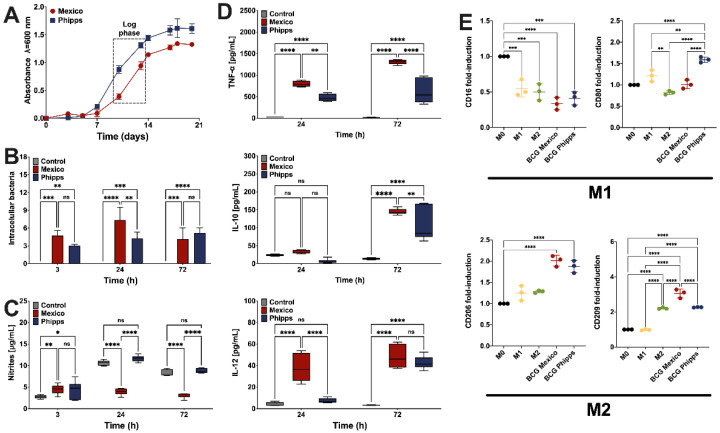
The bacterial growth and the immune characterization. For the growth curves of BCG strains: (**A**) the logarithmic (log) phase was determined at days 10–11 for both strains and is indicated in a dotted rectangle. The survival of the BCG strains in THP-1 macrophages (**B**) was tested at 3, 24 and 72 h postinfection. The nitric oxide production at progressive times by macrophages infected with the BCG strains, or in presence of the control, can be seen in (**C**). The concentrations of TNF-α, IL-10 and IL-12 in the supernatants of THP-1 macrophages infected with the BCG strains 24 h and 72 h postinfection, or of noninfected THP-1 cells, is shown in (**D**). The presence of the surface markers CD16 and CD80, or CD206 and CD209, related to M1 and M2 macrophages, respectively, is seen in (**E**). Data is expressed as means ± s.d. M0: M0 macrophages; M1: M1 macrophages; M2: M2 macrophages. * *p* < 0.05; ** *p* < 0.01; *** *p* < 0.001; **** *p* < 0.0001; ns: nonsignificant.

The first step was to characterize the two BCG strains. Although similar growth curves were observed for both strains, the log phase was achieved at days 11 and 10 for the M.BCG and P.BCG strains, respectively (Figure 1A). Next, the total numbers of intracellular bacteria were quantified 3, 24 and 72 h postchallenge in THP-1-derived macrophages, and only at 24 h was a significant difference between strains seen, with more intracellular bacteria in the Mexico strain (Figure 1B). Using the same time points, NO concentrations were quantified in infected macrophages, and a significant reduction in NO levels was seen when the Mexico strain was present. No significant differences were evident between the control and the Phipps strain at 24 and 72 h, and the NO concentrations were comparable in all the conditions between 24 and 72 h (Figure 1C). Next, cytokines were quantified at 24 and 72 h after the infection, and the results showed that the Mexico strain induced an increase in the secretion of the inflammatory cytokines TNF-α and IL-12. This effect was similar between 24 and 72 h. The same strain also significantly increased the production of the modulatory cytokine IL-10, but only at 72 h postinfection (Figure 1D). Since we saw in the Phipps strain that the levels of TNF-α were comparable between 24 and 72 h, and that no significant differences were evidenced at 72 h postinfection in IL-12 concentration between both strains, we decided to only analyze 24 h postinfection.

Our next step was to characterize the surface markers of the infected macrophages to determine whether the internalized bacteria could modulate a shift in the affected immune cells. First, we evaluated the capacity of our noninfected THP-1 cells to respond to positive controls that induce M1 and M2 phenotypes. Therefore, we quantified in supernatants of THP-1 cells (monocytes), as well as from M0, M1 and M2 macrophages derived from THP-1 cells, the presence of interferon (IFN)-γ, TNF-α, IL-1β and IL-10. We confirmed that unlike monocytes and M0 macrophages, M1 increased the levels of IFN-γ and TNF-α, and M2 macrophages produced more IL-10 (Figure S1A). Next, we infected the THP-1-derived macrophages with one of the two strains and gated them in order to select only the CD11b^+^/CD14^+^ population (Figure S1B). The results showed that the BCG Phipps strain was superior to induce the expression of the M1 marker CD80 in infected macrophages, and the Mexico strain promoted the presence of the CD209 marker of M2 macrophages (Figure 1E). 

Taken together, the results suggest that the BCG strain is able to maintain itself within infected macrophages by reducing the expression of nitrites and increasing the levels of IL-10 over time, shifting the cells into a M2 phenotype. On the other hand, the M1 phenotype related to BCG Phipps infection could be related with the promotion of the production of nitrites. To better understand this phenomenon, we decided to perform microarray analysis on THP-1-derived macrophages infected for 24 h with either strain.

### 2.2. Gene Expression Differences

**Figure 2:**
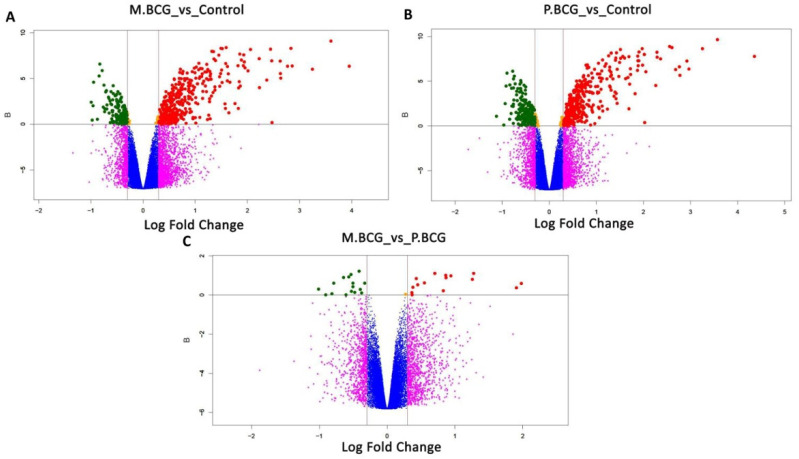
Volcano plot displaying differentially expressed genes in THP-1-derived macrophages: (**A**) Mexico BCG strain vs. control; (**B**) Phipps BCG strain vs. control; (**C**) Mexico BCG strain vs. Phipps BCG strain. B-statistic values are plotted against the log fold change (base 2). A B-statistic of zero corresponds to a 50–50 chance that the gene is differentially expressed (gray line). On the x axis, values outside the red lines represent log fold changes of ≥ 0.3 between M.BCG and P.BCG. Red and green dots represent over- and subexpressed genes, respectively.

**Figure 3:**
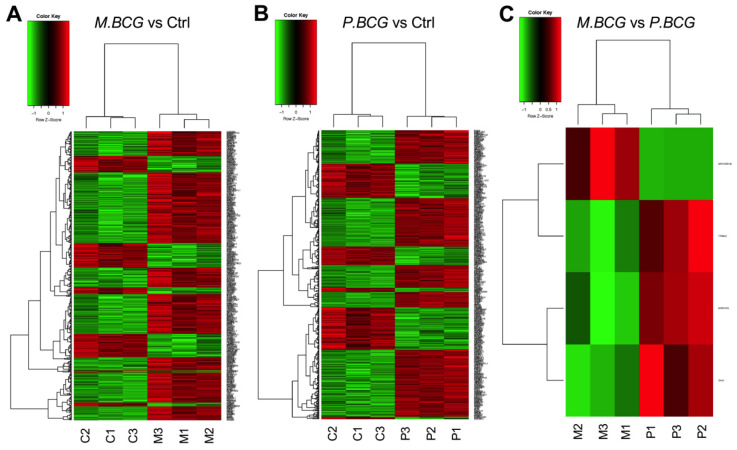
Heatmap representation of differentially expressed genes in THP-1-derived macrophages: (**A**) M.BCG vs. Ctrl; (**B**) P.BCG vs. Ctrl; (**C**) M.BCG vs. P.BCG. Negative z-score indicates subexpressed genes (green), and positive z-score indicates overexpressed genes (red). Genes without significant expression are marked in black. The dendrogram on the left shows the relationship between the genes. M.BCG: *Mycobacterium bovis* Mexico; P.BCG: *Mycobacterium bovis* Phipps; Ctrl: control.

After infection for 24 h with either the M.BCG or P.BCG strains, the THP-1-derived macrophages were subjected to microarray evaluations. The DEGs identified in macrophages infected with the BCG strains were compared against a control group of noninfected macrophages, as shown in Figure 2 and Figure 3. The macrophages infected with M.BCG had 121 downregulated and 337 upregulated genes (Figure 2A), whereas those infected with P.BCG demonstrated 166 downregulated and 304 upregulated genes (Figure 2B) compared with the control macrophages. Four DEGs were identified when comparing M.BCG- and P.BCG-infected macrophages, including three that were upregulated in M.BCG-infected macrophages and one that was upregulated in P.BCG-infected macrophages with a B score ≥ 0 (Figure 2C). Then, heatmaps were generated to compare the expression patterns between each of three biological replicates consisting of macrophages infected with either M.BCG, P.BCG or the control samples. A clear hierarchical separation could be observed in the gene expression patterns of the control group and those in response to infection with M.BCG Mexico (Figure 3A) and P.BCG (Figure 3B). However, the expression profile of M.BCG compared with that of P.BCG showed a small number of DEGs, including the genes *MPHOSPH8*, *MIR31HG*, *SAA1* and *TRIML2* (Figure 3C).

### 2.3. Proinflammatory Signaling Pathways in THP-1-Derived Macrophages in Response to BCG Mexico and Phipps

We investigated the categories of genes that were upregulated in the infected macrophages. A gene set enrichment analysis (GSEA) performed on the expression profile induced by M.BCG infection demonstrated the significant enrichment of cytokine-mediated signaling pathways, including the response to IFN-γ and the positive regulation of leukocyte migration, which obtained nominal *p*-values (NOM *p*-value) < 0.05 (Table 1). The NOM *p*-value represents the probability under the null distribution of obtaining an enrichment score (ES) value that is as strong or stronger than that observed for an experiment under the permutation-generated null distribution. A gene ontology (GO) enrichment analysis showed 445 transcripts that were functionally relevant and clustered with Kyoto Encyclopedia of Genes and Genomes (KEGG) pathways and were primarily associated with influenza A, TNF and chemokine signaling pathways (Table 2). A GSEA performed on the expression profile induced by P.BCG also indicated the significant enrichment of cytokine-mediated signaling pathways. Other enriched pathways were also inflammatory-mediated pathways, such as the response to IFN-γ (Table 3). The GO enrichment analysis revealed 452 transcripts associated with KEGG pathways for influenza and both the TNF and nuclear factor κB (NF-κB) signaling pathways (Table 4). Therefore, our results demonstrated comparable proinflammatory signaling profiles in macrophages infected with either M.BCG or P.BCG.

**Figure 4:**
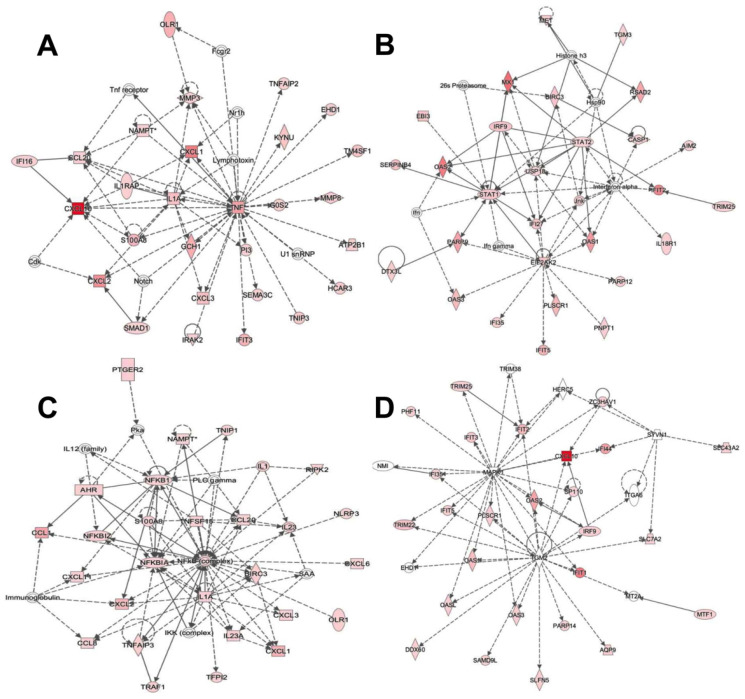
Networks induced by the BCG strains in THP-1-derived macrophages. Networks of functional: (**A**) dermatological diseases and conditions, cell-to-cell signaling and interaction, cellular movement; and (**B**) antimicrobial response, inflammatory response, cell signaling related to M.BCG vs. Ctrl. Network of functional: (**C**) inflammatory response, connective tissue disorders, inflammatory disease; and (**D**) antimicrobial response, inflammatory response, dermatological diseases and conditions related to P.BCG vs. Ctrl. Color codes: red, overexpressed; green, subexpressed. The color intensity indicates the degree of expression. Encoding form: rectangle—nuclear receptor ligand-dependent; oval—transcription regulator; rhombus—enzyme; circle—other.

**Figure 5:**
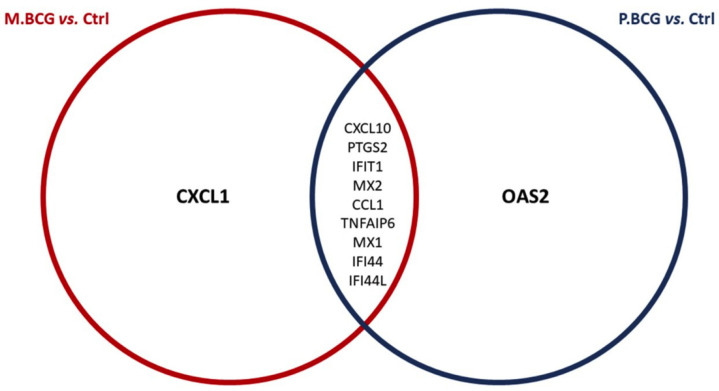
Venn diagram representing the differentiated genes within sets corresponding to the two IPA predictions. The intersections correspond to genes appearing in more than one prediction. Red circle: M.BCG vs. Ctrl; Blue circle: P.BCG vs Ctrl; M.BCG: *Mycobacterium bovis* Mexico; P.BCG: *Mycobacterium bovis* Phipps strain; Ctrl: control.

To understand the functional relationships between the identified DEGs in each strain within the context of biological processes, pathways and networks, Ingenuity Pathway Analysis (IPA) core analyses were performed to obtain a high-score network (score > 15). These scores were derived from *p*-values, which indicated the probability that their appearance in the network was due to background noise. The IPA analysis induced by M.BCG indicated that within the “dermatological diseases” network, the DEGs were significantly related to antimicrobial responses, inflammatory responses, immunological diseases, and connective tissue disorders. A total of 10 upregulated genes were identified (*CXCL10*, *PTGS2*, *IFIT1*, *MX2*, *CCL1*, *TNFAIP6*, *IFI44L*, *MX1*, *IFI44* and *CXCL1*), and an additional 10 downregulated genes were detected (*SUCNR1*, *CYSLTR1*, *TREM2*, *HEY2*, *CD109*, *NRGN*, *DTL*, *VAT1L*, *CD180* and *CDC20*). The networks with the highest scores (score = 46) consisted of (Figure 4A) dermatological diseases and conditions, cell-to-cell signaling and interaction and cellular movement and (Figure 4B) antimicrobial response, inflammatory response and cell signaling. The IPA analysis induced by P.BCG revealed 82 molecules that were identified and grouped into inflammatory response and disease, dermatological diseases and related conditions, antimicrobial response and connective tissue disorders. A total of 10 upregulated genes were identified (*CXCL10*, *PTGS2*, *IFIT1*, *IF144L*, *MX2*, *MX1*, *TNFAIP6*, *IFI44*, *CCL1* and *OAS2*), and no downregulated genes were identified. When these genes were grouped into networks, the networks with the highest scores (score = 43) and were identified as (Figure 4C) inflammatory response, connective tissue disorders and inflammatory disease and (Figure 4D) antimicrobial response, inflammatory response and dermatological diseases and related conditions. Within the two IPA predictions, 9 DEGs associated with innate immune function were observed in THP-1-derived macrophages infected with both M.BCG and P.BCG compared against control macrophages (Figure 5).

### 2.4. Analysis of Gene Expression Patterns Compared between BCG Mexico and BCG Phipps

The analysis of the gene expression patterns induced by M.BCG and P.BCG revealed 31 DEGs between the two conditions, including 15 upregulated and 16 downregulated genes in M.BCG-infected macrophages compared to P.BCG-infected macrophages. According to GSEA, 1562 of the 4479 generated clusters containing genes expressed in the M.BCG group were related to the immune response, with significant enrichment and a false-positive rate < 25% (False Discovery Rate [FDR] *q*-value < 0.25). These pathways involved the regulation of cytokines such as IL-1, IL-10, IL-12 and TNF; phosphoprotein binding and phosphoprotein phosphatase activity. All the referred pathways were significantly enriched in macrophages infected with M.BCG, as compared to macrophages infected with P.BCG (Table 5).

**Figure 6:**
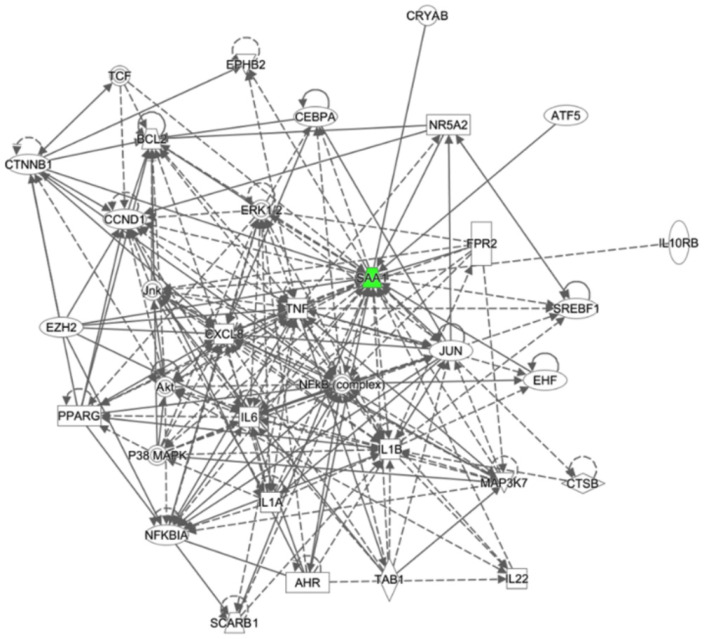
Network of tissue development, gene expression and cellular development related to M.BCG vs. P.BCG. Color codes: green, subexpressed. The color intensity indicates the degree of expression. Encoding form: rectangle—nuclear receptor ligand-dependent; oval—transcription regulator; rhombus—enzyme; circle—other.

The network analysis of the biological interactions among the DEGs identified between M.BCG- and P.BCG-infected macrophages revealed that cancer was the primarily associated pathology. One cancer-associated gene (*MPHOSPH8*) was upregulated in samples infected with M.BCG relative to those infected with P.BCG, whereas three genes were downregulated in M.BCG (*MIR31HG*, *SAA1*, and *TRIML2*). The network with the top score (score = 2) included tissue development, gene expression and cellular development (Figure 6). This network includes pathways associated with vesicle-mediated transport and activated TLR4 signaling. GO annotations related to the *SAA1* gene included chemoattractant activity.

## 3. Discussion

The innate immune response is critical for protection against infections and is involved in the protective response triggered by vaccines. The BCG vaccine offers 50% protection against pulmonary tuberculosis. Several explanations may exist for this phenomenon. One possibility is the different compositions of the cell walls in each BCG strain [23,24,25], which can affect their capacities to be phagocytosed by macrophages, hampering bacterial pathogenesis [26,27,28].

In this study, the initial characterization of the immune response induced by the macrophage challenged with the strains M. BCG and P. BCG yielded results that were consistent with previous reports. The THP-1 cell line is an alternative study model that serves as a support to understand various cellular responses without assuming that the results are the same as those of macrophages obtained live from patients [20]. The spectrophotometric absorbance values observed for each strain over time, which were used to estimate the log phase for maximal growth, were similar to those reported by other authors examining different BCG strains [29,30]. The differential amount of intracellular bacteria we found over time could be associated with the phenotypic characteristics of each strain and could explain both the phagocytosis process and the immune response [23,24,25,31]. Indeed, BCG Phipps possesses an 86-carbons methoxymycolic acid, which is longer that that found in BCG Mexico of 84-carbons [23]. Although the presence of this acid could be related to the differential recognition of BCG Phipps, additional studies to decipher the implicated structures in the response are needed. Additionally, the sequencing of the BCG Mexico genome has revealed the existence of specific polymorphisms for this particular strain, such as in the RDMex02 region where a deletion of 218 amino acids of the *fadD23* gen has been reported, altering a preserved region of the protein that includes two transmembranal domains [31]. Since this gene codifies for a probable fatty acid acyl-CoA ligase involved in the lipid degradation and in the production of sulpholipids, its disruption could increase the association of the BCG strain and the infected macrophage.

Infection with either M.BCG or P.BCG stimulates the expression of the proinflammatory molecule NF-κB in the host cell [23], indicating the promotion of effector molecules as a response to infection. In agreement with our previous in vivo report in mice, infection with the P.BCG strain was associated with reduced IL-10 expression [19]. In addition, the upregulation of both IL-12 and TNF-α expression has been previously described for human monocyte-derived macrophages stimulated with the BCG Tokyo strain for up to 24 h [32]. Our results showed that the concentration of NO induced by M.BCG infection for 24 h did not increase when compared with that induced by P.BCG infection. Hayashi et al. showed that in both A549 human cells of alveolar epithelial origin and in mice bone marrow cells, NO production increased after the cells were incubated with P.BCG [23]. Although their quantifications were performed 48 h after the challenge, the NO concentrations reported by the previous study were similar to those observed in our study at 24 h following infection. Thus, after observing similar and expected results with each strain relative to the previously reported results, we pursued a transcriptional analysis of the infected macrophages.

To our knowledge, this is the first study comparing the responses of macrophages infected with two different BCG strains at the transcriptional level. We analyzed the transcriptional signatures of macrophages infected with M.BCG and P.BCG and analyzed the results using the enrichment analysis tools GSEA, Database for Annotation, Visualization, and Integrated Discovery (DAVID) and IPA. GSEA produced a set of five consistently enriched functions induced by infections with both strains: viral response, viral defense, cytokine-mediated signaling pathway, cellular response to IFN-γ and response to type I interferon. The DAVID analysis identified three functional categories for both strains: the TNF signaling pathway, the NF-κB signaling pathway and the chemokine signaling pathway. DAVID also identified the functional pathway of cytokine–cytokine receptor interaction in M.BCG infection, whereas the P.BCG infection included the NOD-like receptor signaling pathway and the Toll-like receptor (TLR) signaling pathway. In 2015, Rienksma et al. reported that after 24 h of infection with the Danish BCG SSI 1331 strain, THP-1 cells were enriched in multiple pathways associated with the innate immune responses, with the three most upregulated pathways including those associated with IFN-α/β signaling, IFN-γ signaling, and the retinoic acid-inducible gene I (RIG-I)- melanoma differentiation-associated protein five (MDA5)-mediated induction of the IFN-α/β pathways [33]. An additional report indicated that 4 h of infection in THP-1 cells was sufficient to stimulate the expression of multiple miRNAs related to the proinflammatory response, including miR-146a [34]. The miR-146a is quickly activated in human monocytes to target the TNF receptor-associated factor six (TRAF6), which is a strong modulator of the TLR activity within the innate immune response [35]. In accordance with previous studies, in our work, both the M.BCG and P.BCG strains induced the expression of genes associated with the interferon and TNF signaling pathways in THP-1 cells. The similar patterns of innate immune response-associated gene expression induced by both strains suggested that their behaviors were consistent with our previous findings, in which we compared the effectiveness of 10 BCG strains in a mouse model of pulmonary tuberculosis. In that study, BALB/c mice were subcutaneously vaccinated and 2 months later challenged with the *Mycobacterium tuberculosis* H37Rv strain by intratracheal injection. After 2 and 4 months, a delayed-type hypersensitivity (DTH) response, the degree of pneumonia-affected lung tissue, CFU, T cell count, and cytokine expression (IL-2, IL-4, IL-10, and IFN-γ) were determined. Differential protective effects across the diverse BCG strains were found with P.BCG resulting in the largest and most persistent reduction in CFU counts and pneumonia at both 2 and 4 months after challenge. This protection was accompanied by a reduction in IL-10-producing T cells. Contemporary BCG strains, which are characterized by the presence of a mutated or upregulated *inhA* gene that confers resistance against ethionamide and isoniazid, induced a wide range of protective effects in this animal model [19].

As an approach to integrate the previous results, we conducted an IPA evaluation. IPA has previously shown its capacity to identify innate immune signaling pathways in macrophages stimulated with mycobacteria [36] and those that involve pattern-recognizing molecular components [37]. The IPA analysis showed similar biological interactions induced by M.BCG and P.BCG. The DEGs in both groups were categorized into four networks: antimicrobial response, inflammatory response and disease, dermatological diseases and conditions and connective tissue disorders. M.BCG stimulated the upregulation of *CXCL1*. CXCL1 recruits and activates neutrophils, is associated with protection in *H. pylori* and meningitis infections and is involved in the signaling pathways of G protein-coupled receptors [38]. The P.BCG infection stimulated the upregulation of *OAS2,* which is related to the INF-γ and NOD2 signaling pathways [39], and which is associated with restriction of the replication of intracellular mycobacteria and promotion of cytokine secretion [40]. We observed the downregulation of 10 genes by M.BCG only (*SUCNR1*, *CYSLTR1*, *TREM2*, *HEY2*, *CD109*, *NRGN*, *DTL*, *VAT1L*, *CD180* and *CDC20*), which among other pathways, are associated with Notch signaling, succinate recognition, Th1/Th2 balance and anti-inflammatory responses. Their downregulation could imply reversion of the Th1/Th2 imbalance related with tuberculosis progression [41], prevention of anti-inflammatory responses in macrophages [42,43] and promotion of TLR activity [44]. When the IPA analysis was performed to compare gene expression in response to M.BCG relative to the response to P.BCG, we found that the only upregulated gene in M.BCG was *MPHOSPH8*, which encodes a protein component of heterochromatin and whose function is related to epigenetic repression and to TNF response in monocytes [45]. Three additional genes were downregulated in M.BCG relative to P.BCG (*MIR31HG*, *SAA1* and *TRIML2*). *MIR31HG* is a long-noncoding RNA associated with hypoxia that forms a complex with hypoxia-inducible factor-1A [46]. *SAA1* encodes a member of the serum amyloid A family that is a major acute-phase protein and is highly expressed in response to inflammation and tissue injury [47]. *TRIML2* encodes a protein that increases the transactivation of a subset of p53 target genes associated with prolonged DNA damage and apoptosis [48]. The expression of these genes by M.BCG leads us to theorize that M.BCG promotes macrophage proliferation by decreasing hypoxia and apoptosis. 

Very recently, it has been reported in vitro assays with infected monocytes that the expression of lncRNAs is BCG strain-dependent. In such case, these molecules could play an important role in the pathogenesis of tuberculosis and could be used as potential biomarkers for this disease, as well as therapeutic targets [49]. More specifically, the dysregulation of the lncRNA MIR31HG has been described in immune-altered environments such as cancer, modifying the cell survival by downregulating the expression of components of the Epidermal Growth Factor Receptor [50]. In rheumatoid arthritis, it downregulates the PI_3_K/AKT pathway to modulate the proinflammatory responses of associated macrophages, altering the production of IL-6, IL-8 and TNF-α [51]. Additionally, MIR31HG has been described as a regulator of the components of the senescence-associated secretory phenotype (SASP) through the modulation of IL-1A translation in BRAF-induced senescent cells. In fact, when *MIR31HG* is depleted, the cells fail to produce the innate immune mediators IL-6 and CXCL1 and are unable to migrate in transwell assays [52]. The phenomenon of senescence has been related to oncogenic transformation, and it is not surprising that high expression of MIR31HG is linked to enrichment in TNF and type-I interferon [53]. In THP-1 cells, the presence of exogenous pathogen-associated molecular patterns (PAMPs) engages the DNA sensor cyclic GMP-AMP synthase, which then enhances type-I interferon responses [54]. Among other targets, type-I interferon promotes the tumor suppressor TRIML2, which also elicits antiviral and antibacterial activities [55]; it participates in the correct development of macrophages and in the expression of nitric oxide and of the major histocompatibility complex II to present antigens [56]. An additional type-I interferon target is MPHOSPH8 [57], which belongs to the Human Silencing Hub (HUSH) complex, and it regulates the chromatin structure. In lung carcinoma mice models, it has been reported that low expression levels of MPHOSPH8 are linked to sensitization to anti-PD-1/CTLA-4 therapies [58], implying a feasible association of this epigenetic regulator and the suppression of the immune evasion. To our knowledge, our use of a THP-1 model infected with either P.BCG or M.BCG marks the first time that a differential expression in *MIR31HG*, *TRIML2* and *MPHOSPH8*, three genes which, as indicated above, act as immune modulators, and could explain the production of cytokines and the cellular activation has been reported. Regarding SAA1, stimulated THP-1 cells have proven to increase the expression of *SAA1* [59], and the high expression of *SAA1* in activated human monocytes has been associated with upregulation in *IL1A*, *IL1B* and *IL6* [60]. Therefore, the modulation of *MIR31HG*, *SAA1* and *TRIML2* could be linked to the M2 polarization seen in the infected macrophages with the Mexico strain.

Taken together, these data open the possibility of a regulatory network involving type-I interferon and the four reported genes in THP-1-infected cells. Surprisingly, in the microarray data obtained in the comparative M.BCG vs. P.BCG, we did not identify differentially expressed genes such as *TNF*, *IL10* and *IL12*, as well as genes involved in pathways associated with nitric oxide. A plausible explanation for this result could be transcript buffering, which secures an adjustment in mRNA synthesis and degradation to ensure a balance in total cellular mRNA depots [61]. Since close to half of all the protein concentration variation is regulated at the translational level [62], and because the translation of *TNF*, *IL10* and *IL12* are modulated by DEAD-box helicases such as eIF4A in monocytes [63], this statement is possible. However, to correctly identify alterations in translational regulation, further experiments such as polysome profiling are required to explore mRNA efficiently bound to ribosomes. With our current results, we could hypothesize that the reported DEGs, therefore, suggest fine physiological changes in the infected macrophages. It is possible that the DEGs we found could be related to senescence, like *MIR31HG*, which could be determinant for the survival of the macrophage and the bacteria, and for the adaptive response as well. Overall, the differential expression of *MPHOSPH8*, *MIR31HG*, *SAA1* and *TRIML2* could show us the subtle changes that occur in the infection between M.BCG and P.BCG. We also identified the stimulated expression of TLR members, of NOD, of TNF, of chemokines, of cytokines and of type I interferons, which, together with the activation of NF-κB, indicate an inflammatory environment.

In conclusion, our data suggested that both strains stimulated a proinflammatory response at the levels of cytokines and transcripts. It should be noted that the inflammatory response of M. BCG was observed more pronounced compared to P. BCG at 24 hours of analysis. These data seem to indicate that M. BCG takes greater control of the macrophage response since it responds with greater intensity to the infection. On the other hand, P. BCG appears to be controlled from the beginning of the infection, secondary to the upregulation of the *OAS2* gene that controls intracellular replication. The changes observed at this time may vary with a longer time of infection of the macrophage with BCG strains, secondary to the plasticity and reversibility of the macrophage’s polarization capacity. However, longer time analyzes are required, as well as the verification that changes at the gene level translate into changes in protein expression.

## 4. Materials and Methods

### 4.1. BCG Strains

Marcel Behr from the McGill International Tuberculosis Centre (Montréal, QC, Canada) kindly donated the BCG Phipps strain. The BCG Mexico strain was obtained from the Ministry of Health (México).

### 4.2. Bacterial Growth Curve

The BCG strains were grown separately in Middlebrook medium (Difco Laboratories, Detroit, MI, USA) supplemented with 10% ADC (5% bovine serum albumin [fraction V], 2% dextrose, and 0.005% catalase) and 0.05% Tween 80 and were incubated under conditions of constant agitation at 37°C with 5% CO_2_. Optical density was measured every 24 h with a Magellan spectrophotometer (TECAN GENios Plus, Grödig, Austria) until the bacterial concentration reached 1 × 10^8^ bacteria/mL.

### 4.3. Bacterial Cultures

BCG strains were grown as described for the bacterial growth curve experiments. When the bacteria reached a concentration of 1 × 10^8^ bacteria/mL, the CFU was determined. Bacteria were stored in aliquots of 1 × 10^7^ bacteria/mL at −70 °C until use. The viability of each BCG strain was determined by measuring the bacterial ATP content using an ATP Kit SL Luminescent Assay (BioThema AB, Handen, Sweden), and the determination of CFU was performed after 3 weeks of growth on incubation plates consisting of Middlebrook 7H10 agar (Difco Laboratories, Detroit, MI, USA) supplemented with 10% OADC (oleic acid-albumin-dextrose-catalase acid) (Sigma–Aldrich; Merck Millipore, Darmstadt, Germany).

### 4.4. Human Cell Line

The THP-1 cell line (ATCC, TIB-202 (Thermo Fisher Scientific Inc, MA, USA) of human leukemia monocyte origin was maintained in RPMI-1640 medium (Sigma–Aldrich; Merck Millipore, Darmstadt, Germany) supplemented with 10% fetal bovine serum (Sigma–Aldrich; Merck Millipore, Darmstadt, Germany) at 37 °C and 5% CO_2_. After reaching confluence of 1 × 10^6^ THP-1 cells/well in 12-well plates, monocytes were stimulated using 50 ng/mL of Phorbol 12-myristate 13-acetate (PMA) (Sigma–Aldrich; Merck Millipore, Darmstadt, Germany) for 48 h [64]. Later, macrophages were cultivated in medium Differentiation into M1 or M2 phenotypes was performed in noninfected macrophages, as previously described [65]. Briefly, macrophages were incubated for 24 h with 20 ng/mL of human IFN-γ (PeproTech, NJ, USA) plus 100 ng/mL LPS (Sigma–Aldrich; Merck Millipore, Darmstadt, Germany), or with 20 ng/mL of human IL-4 plus 20 ng/mL of human IL-13 (both from PeproTech, NJ, USA), to polarize into M1 or M2 phenotypes, respectively. The polarization of macrophages was confirmed by flow cytometry. Briefly, cells were incubated for 24 h as explained before, and the concentrations of IFN-γ, TNF-α, IL-1β and IL-10 were measured in a flow cytometer, testing either supernatants from nontreated THP-1 cells (monocytes) or from each one of the polarized macrophages following the manufacturer’s instructions (Milliplex Map Human Human Cytokine/Chemokine Magnetic Bead Panel; Millipore) and Veinalde R. et al. [66] (Figure S1). 

### 4.5. Macrophage Infection with the BCG Strains

The bacterial culture was resuspended, and macrophages were infected at a 1:10 ratio (1 × 10^6^ THP-1 cells with 1 × 10^7^ bacteria) with each BCG strain in 12 well-plates. At 3 h postchallenge (time 0), macrophages were washed, and nonphagocytosed bacteria were removed by incubation with 200 µg/mL amikacin for 2 h, which represents a previously reported bactericidal concentration [67]. Macrophages at 3 h, 24 h and 72 h postchallenge were lysed with a lytic solution (0.15 M NaCl, 0.0013 M EDTA, 0.05 M Tris, 0.5% Triton X-100, 0.5% SDS), and the number of intracellular bacteria was calculated by quantifying bacterial ATP contents using the ATP SL Luminescent Assay Kit (BioThema AB, Handen, Sweden ) at 560 nm in the Magellan spectrophotometer. Quantitative culture to determine bacterial numbers was performed using 10-fold serial dilutions on Middlebrook 7H10 agar plates supplemented with 10% OADC.

### 4.6. Nitrite Quantification

NO secreted into the supernatant by infected THP-1-derived macrophages was evaluated at times 0 (3 h postchallenge), 24 h and 72h postchallenge with the Measure-iT Nitrite Assay Kit (Molecular Probes), according to the manufacturer’s instructions. NaNO_2_, at 200 µM/100 µL, was used as a control. Fluorescence was measured at 360/430 nm in a Magellan spectrophotometer.

### 4.7. Cytokine Quantification

IL-10, IL-12 and TNF-α were measured in the supernatant of THP-1-derived macrophages 24 h and 72 h after infection with the BCG strains. Noninfected macrophages were used as controls for comparison. The concentration of each cytokine was quantified by enzyme-linked immunosorbent assay (ELISA) using commercial sandwich assays and performed according to the manufacturer’s instructions (DuoSet ELISA Development System (R&D Systems, Minneapolis, MN, USA)).

### 4.8. Immunophenotype of the Infected Macrophages

Noninfected M0, M1 and M2 macrophages and infected macrophages by either the BCG Mexico or BCG Phipps strains were tested in order to identify the immune phenotype induced by each strain. Briefly, 1 × 10^6^ noninfected M0, M1 or M2 macrophages were collected after the cytokine stimulation, as described in Section 4.4; for infected macrophages, 1 × 10^6^ THP-1 cells were infected with 1 × 10^7^ bacteria, as described in Section 4.5. Next, macrophages were collected, and the CD11b^+^/CD14 (BioLegend, San Diego, CA, USA) cellular population was selected using the flow cytometer Attune Acoustic Focusing Cytometer (Life Technologies, Carlsbad, CA, USA). The surface markers CD16, CD80, CD206 and CD209 (BioLegend, CA, USA) [68,69,70] were tested on this population using the fluorochromes indicated in Table 6 to identify the immunophenotype of the macrophages. The resulting data was analyzed with FlowJo v10, and the expression data was normalized versus M0 macrophages.

### 4.9. RNA Isolation from Macrophages, Evaluation of RNA Integrity, and Microarray Assay

THP-1-derived macrophages were infected with either M.BCG or P.BCG at a 1:10 ratio, using noninfected macrophages as a negative control. After 24 h, macrophages were homogenized, and total RNA was recovered using the TRIzol reagent (Sigma–Aldrich; Merck Millipore, Darmstadt, Germany), following the manufacturer’s instructions. The RNA concentration and purity were determined using a NanoDrop 1000 (Thermo Scientific, Waltham, MA, USA). RNA integrity was evaluated by electrophoresis using 1% agarose gels embedded in TE buffer (100 V, 25 min) (High-Performance UV Transilluminator, UVP). A Eukaryote Total RNA Nano Chip (Agilent Technologies, Palo Alto, CA, USA) was used, following the manufacturer’s instructions. The chips were read with an Agilent 2100 Bioanalyzer (Agilent Technologies, Palo Alto, CA, USA ), and an RNA integrity number (RIN) value with a mean of 8 was accepted. RNA aliquots (20 μL/each) were evaluated with expression microarrays in the Genotyping and Expression Analysis Unit (INMEGEN, UNAM), using the Gene Chip^®^Human Gene 1.0ST Array (Affymetrix, Santa Clara, CA, USA).

### 4.10. Microarray Gene Expression Analysis

Samples were classified into three groups: (1) BCG Mexico-infected macrophages (M.BCG), (2) BCG Phipps-infected macrophages (P.BCG) and (3) noninfected macrophages as a control (Ctrl). All possible pairwise comparisons between the three groups generated three contrasts of interest: M.BCG vs. Ctrl, P.BCG vs. Ctrl and M.BCG vs. P.BCG. We performed a low-level data analysis in which the raw microarray data were background-corrected using the Robust Multi-array Average method and normalized using the quantile normalization approach [71,72], which were both executed in R, V3.1.3 (http://www.cran.r-project.org, accessed on 4 March 2022). DEGs were determined by fitting a linear model to each gene using the Limma package with an empirical Bayesian approach [73,74]. The correction for multiple hypotheses was applied by controlling the FDR. Genes were selected as differentially expressed based on |log fold-change| ≥ 0.3, and significance was determined based on a B-statistic ≥ 0 with associated FDR adjusted *p*-values ≤ 0.01. The expression matrix created was employed for enrichment analyses and the selection of DEGs. Three tools were used to evaluate functional properties and perform pathway analyses for the DEGs. We used gene annotation enrichment analysis within the set of significant genes, employing the DAVID bioinformatics tool, V6.7 (http://david.abcc.ncefcrf.gov/, accessed on 4 March 2022) [75,76]. Enrichment analysis was also conducted using the IPA platform Ingenuity, v.26127183 (Redwood City, CA, USA), and the Gene Set Enrichment Analysis software tool (GSEA) of the Broad Institute of Harvard and M.I.T. [77,78].

### 4.11. Statistical Analysis

All experiments were performed in three independent replicates. The counts of intracellular bacteria, as well as the measurements of nitrites, cytokines and surface markers of infected macrophages were analyzed using a two-way analysis of variance (ANOVA) with Tukey’s correction. Data are presented as the mean and standard deviation. All statistical analyses were conducted using GraphPad Prism Software, v9.0. A *p*-value ≤ 0.05 was considered statistically significant.

## Figures and Tables

**Table 1:** Gene set enrichment analysis in M.BCG compared against the control. ES: Enrichment Score; NES: Normalized Enrichment Score; NOM *p*-val: Nominal *p*-value; FDR *q*-val: False Discovery Rate *q*-value; M.BCG: *Mycobacterium bovis* Mexico strain.

Name of the Pathway	Size	ES	NES	NOM *p*-val	FDR *q*-val
RESPONSE_TO_VIRUS	205	0.76	0	0	0.008
DEFENSE_RESPONSE_TO_VIRUS	132	0.79	0	0	0.012
CYTOKINE_MEDIATED_SIGNALING_PATHWAY	391	0.67	0	0	0.009
RESPONSE_TO_INTERFERON_GAMMA	114	0.76	0	0	0.011
RESPONSE_TO_TYPE_I_INTERFERON	50	0.87	0	0	0.011
NEGATIVE_REGULATION_OF_VIRAL_PROCESS	78	0.81	0	0	0.023
CELLULAR_RESPONSE_TO_INTERFERON_GAMMA	95	0.77	0	0	0.020
POSITIVE_REGULATION_OF_LEUKOCYTE_MIGRATION	104	0.74	0	0	0.020
NEGATIVE_REGULATION_OF_MULTI_ORGANISM_PROCESS	136	0.72	0	0	0.022
IMMUNE_EFFECTOR_PROCESS	393	0.62	0	0	0.020
REGULATION_OF_VIRAL_GENOME_REPLICATION	64	0.78	0	0	0.023
POSITIVE_REGULATION_OF_LEUKOCYTE_CHEMOTAXIS	77	0.76	0	0	0.021

**Table 2:** Enriched gene ontology terms of the transcripts in M.BCG compared against the control. M.BCG: *Mycobacterium bovis* Mexico.

KEGG Pathway	Genes	%	Enrichment	*p*-Value	Benjamini
Influenza A	30	6.74	5.3186	0.000	0.000
TNF signaling pathway	22	4.94	6.4024	0.000	0.000
Chemokine signaling pathway	27	6.06	4.4779	0.000	0.000
Herpes simplex infection	26	5.84	4.3828	0.000	0.000
NF-kappa B signaling pathway	17	3.82	6.0278	0.000	0.000
Measles	20	4.49	4.6388	0.000	0.000
Legionellosis	13	2.92	7.4264	0.000	0.000
Hepatitis C	19	4.26	4.4068	0.000	0.000
Cytokine-cytokine receptor interaction	25	5.61	3.3530	0.000	0.000
Malaria	12	2.69	7.5546	0.000	0.000
Rheumatoid arthritis	15	3.37	5.2582	0.000	0.000
NOD-like receptor signaling pathway	12	2.69	6.7305	0.000	0.000
Pertussis	13	2.92	5.3470	0.000	0.000
Toll-like receptor signaling pathway	15	3.37	4.3653	0.000	0.000

**Table 3:** Gene set enrichment analysis in P.BCG compared against the control. ES: Enrichment Score; NES: Normalized Enrichment Score; NOM *p*-val: Nominal *p*-value; FDR *q*-val: False Discovery Rate *q*-value; P.BCG: *Mycobacterium bovis* Phipps strain.

Name of the Pathway	Size	ES	NES	NOM *p*-val	FDR *q*-val
DEFENSE_RESPONSE-TO-VIRUS	132	0.82	2.98	0.00	0.00
RESPONSE_TO_VIRUS	205	0.77	2.98	0.00	0.00
CYTOKINE_MEDIATED_SIGNALING_PATHWAY	391	0.70	2.89	0.00	0.00
RESPONSE_TO_INTERFERON_GAMMA	114	0.79	2.79	0.00	0.00
CELLULAR_RESPONSE_TO_INTERFERON_GAMMA	95	0.80	2.79	0.00	0.00
NEGATIVE_REGULATION_OF_VIRAL_PROCESS	78	0.83	2.74	0.00	0.00
RESPONSE_TO_TYPE_I_INTERFERON	50	0.89	2.71	0.00	0.00
NEGATIVE_REGULATION_OF_MULTI_ORGANISM_PROCESS	136	0.74	2.70	0.00	0.00
INFLAMMATORY_RESPONSE	401	0.65	2.65	0.00	0.00
IMMUNE_EFFECTOR_PROCESS	393	0.64	2.63	0.00	0.00
POSITIVE_REGULATION_OF_LEUKOCYTE_MIGRATION	104	0.74	2.58	0.00	0.00
INTERFERON_GAMMA_MEDIATED_SIGNALING_PATHWAY	52	0.83	2.57	0.00	0.00

**Table 4:** Enriched gene ontology categories of the transcripts in P.BCG compared against the control. P.BCG: *Mycobacterium bovis* Phipps.

KEGG Pathway	Genes	%	Enrichment	*p*-Value	Benjamini
Influenza A	31	6.86	5.5959	0.000	0.000
TNF signaling pathway	21	4.65	6.2226	0.000	0.000
NF-kappa B signaling pathway	19	4.20	6.8595	0.000	0.000
Herpes simplex infection	26	5.75	4.4625	0.000	0.000
Chemokine signaling pathway	26	5.75	4.3905	0.000	0.000
Legionellosis	15	3.32	8.7247	0.000	0.000
Measles	21	4.65	4.9593	0.000	0.000
Hepatitis C	20	4.42	4.7232	0.000	0.000
Pertussis	15	3.32	6.2818	0.000	0.000
Malaria	12	2.65	7.6920	0.000	0.000
NOD-like receptor signaling pathway	12	2.65	6.8529	0.000	0.000
Toll-like receptor signaling pathway	16	3.54	4.7410	0.000	0.000

**Table 5:** Gene set enrichment analysis in M.BCG vs. P.BCG. ES: Enrichment Score; NES: Normalized Enrichment Score; NOM *p*-val: Nominal *p*-value; FDR *q*-val: False Discovery Rate *q*-value; M.BCG: *Mycobacterium bovis* Mexico strain; P.BCG: *Mycobacterium bovis* Phipps strain.

Name of the Pathway	Size	ES	NES	NOM *p*-val	FDR *q*-val
POSITIVE_REGULATION_OF_INTERLEUKIN_1_SECRETION	22	−0.46	−1.22	0.20	0.64
REGULATION_OF_INTERLEUKIN_1_SECRETION	29	−0.40	−1.13	0.31	0.75
POSITIVE_REGULATION_OF_INTERLEUKIN_1_PRODUCTION	34	−0.34	−0.99	0.46	0.92
REGULATION_OF_INTERLEUKIN_1_PRODUCTION	53	−0.28	−0.89	0.62	1
REGULATION_OF_INTERLEUKIN_12_PRODUCTION	46	−0.26	−0.80	0.77	1
INTERLEUKIN_1_PRODUCTION	15	−0.33	−0.80	0.73	1
REGULATION_OF_INTERLEUKIN_10_PRODUCTION	37	−0.25	−0.75	0.86	1
POSITIVE_REGULATION_OF_INTERLEUKIN_12_PRODUCTION	31	−0.24	−0.68	0.91	1
NEGATIVE_REGULATION_OF_INTERLEUKIN_1_PRODUCTION	17	−0.17	−0.43	0.99	1
REGULATION_OF_INTERLEUKIN_1_BETA_PRODUCTION	43	−0.13	−0.40	1	1
TUMOR_NECROSIS_FACTOR_RECEPTOR_BINDING	25	−0.49	−1.32	0.13	0.55
TUMOR_NECROSIS_FACTOR_RECEPTOR_SUPERFAMILY_BINDING	38	−0.42	−1.22	0.18	0.63
NEGATIVE_REGULATION_OF_TUMOR_NECROSIS_FACTOR_SUPERFAMILY_CYTOKINE_PRODUCTION	35	−0.30	−0.89	0.64	1
REGULATION_OF_TUMOR_NECROSIS_FACTOR_SUPERFAMILY_CYTOKINE_PRODUCTION	87	−0.24	−0.80	0.83	1
TUMOR_NECROSIS_FACTOR_MEDIATED_SIGNALING_PATHWAY	106	−0.19	−0.67	0.98	1
RESPONSE_TO_TUMOR_NECROSIS_FACTOR	208	−0.17	−0.62	1.00	1
REGULATION_OF_TUMOR_NECROSIS_FACTOR_BIOSYNTHETIC_PROCESS	15	−0.25	−0.61	0.93	1
REGULATION_OF_TUMOR_NECROSIS_FACTOR_MEDIATED_SIGNALING_PATHWAY	41	−0.18	−0.54	1.00	1
POSITIVE_REGULATION_OF_TUMOR_NECROSIS_FACTOR_SUPERFAMILY_CYTOKINE_PRODUCTION	52	−0.17	−0.53	1.00	1
PHOSPHOPROTEIN_BINDING	56	−0.21	−0.67	0.95	1
PHOSPHOPROTEIN_PHOSPHATASE_ACTIVITY	152	−0.18	−0.66	0.99	1

**Table 6:** Surface markers of each subpopulation of THP-1-derived macrophages.

Subpopulation	Surface Marker	Fluorochrome
M0	CD11b	APC
	CD14	PE
M1	CD16	APC/Cy7
	CD80	FITC
M2	CD206	FITC
	CD209	APC

## Data Availability

All the data and material are available upon reasonable request to the corresponding author.

## Supplementary Materials

The following supporting information can be downloaded at: https://www.mdpi.com/article/10.3390/ijms23094525/s1.
