# Supplementary figures and images for: RNA Microarray-Based Comparison of Innate Immune Phenotypes between Human THP-1 Macrophages Stimulated with Two BCG Strains
Source: Int J Mol Sci. 2022 Apr 20;23(9):4525. doi: 10.3390/ijms23094525 (PMC9103163; doi:10.3390/ijms23094525)

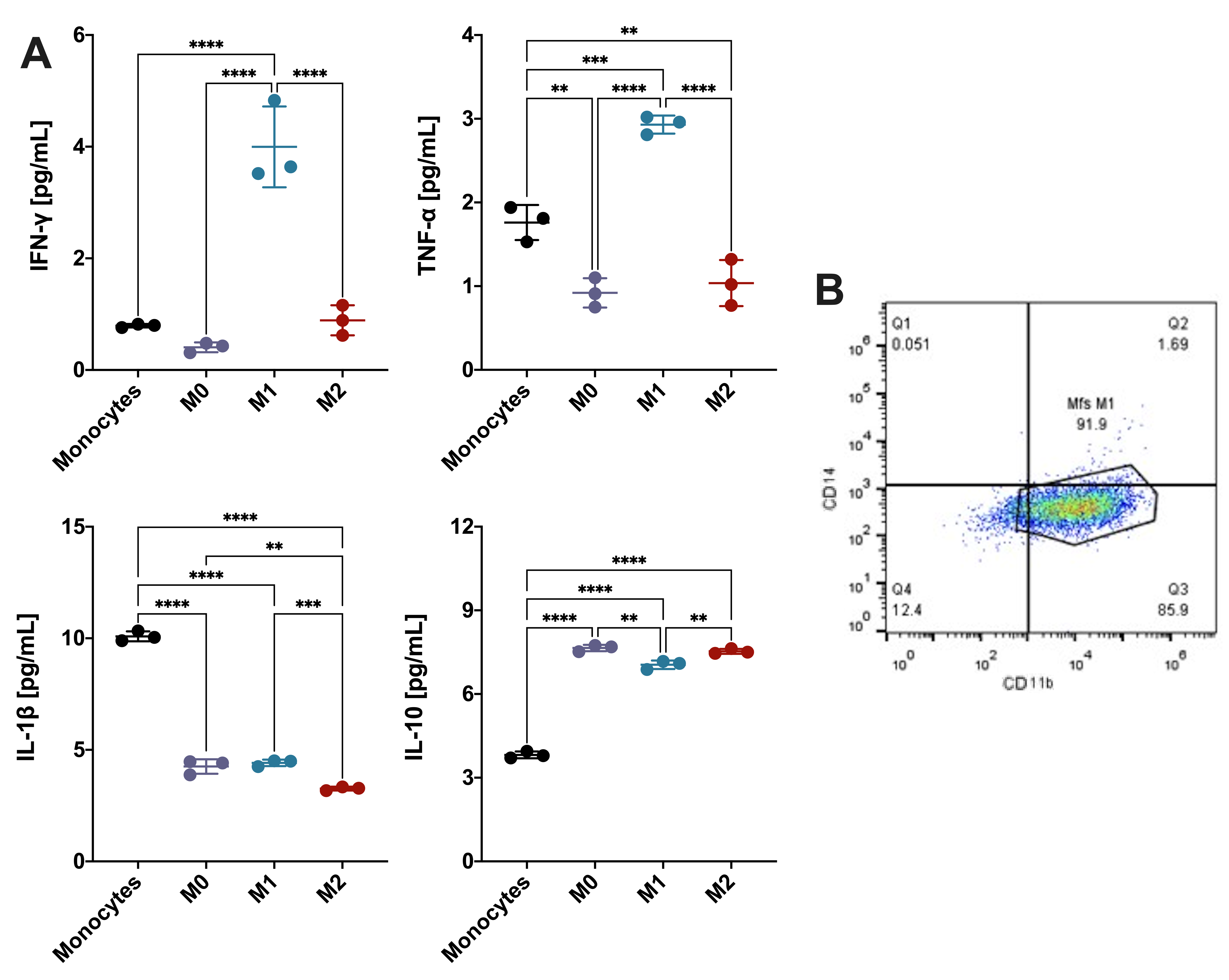

Supplement: Supplementary file 1 [file ijms-23-04525-s001.zip › ijms-1697224-supplementary.tiff]
